# Supplementary figures and images for: GC-AG Introns Features in Long Non-coding and Protein-Coding Genes Suggest Their Role in Gene Expression Regulation
Source: Front Genet. 2020 May 15;11:488. doi: 10.3389/fgene.2020.00488 (PMC7242645; doi:10.3389/fgene.2020.00488)

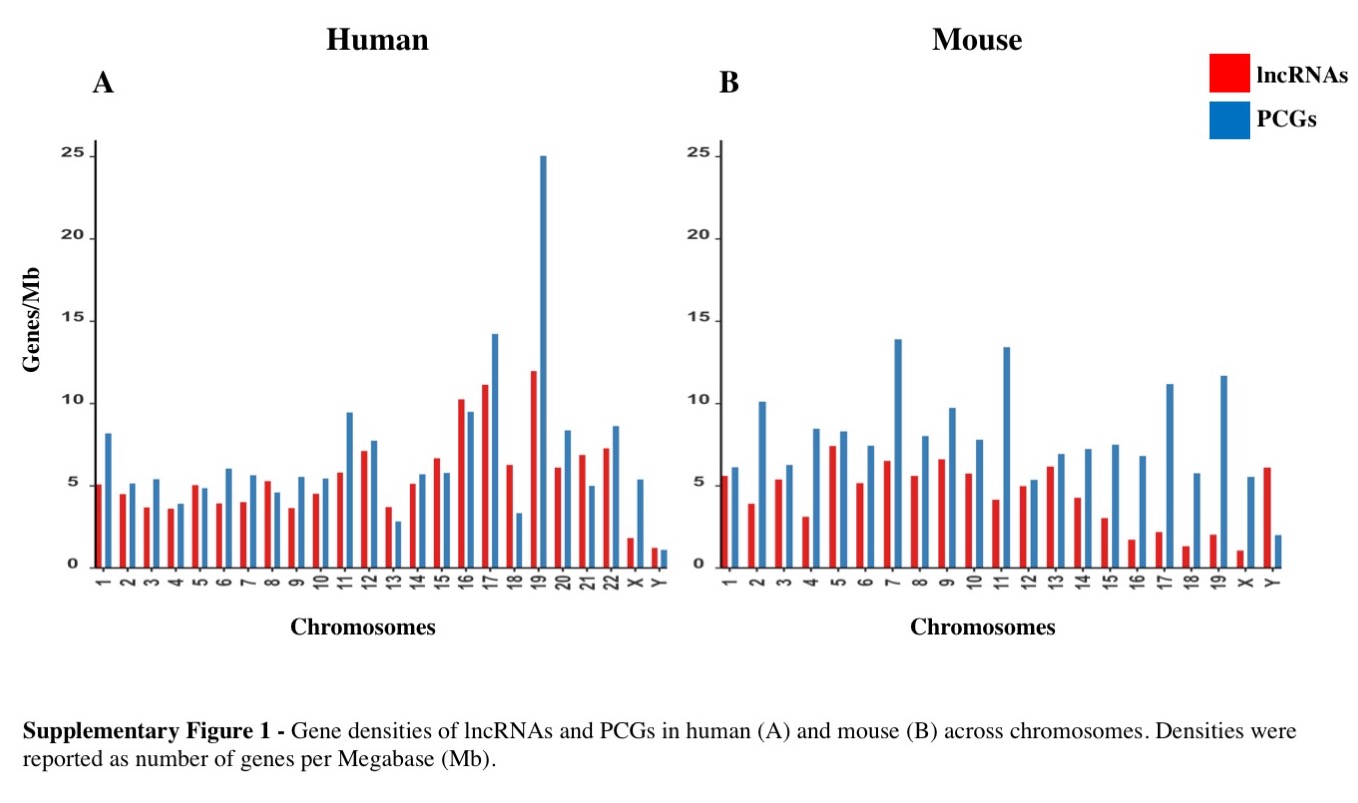

Supplement: Supplementary file 2 [file Image_1.JPEG]

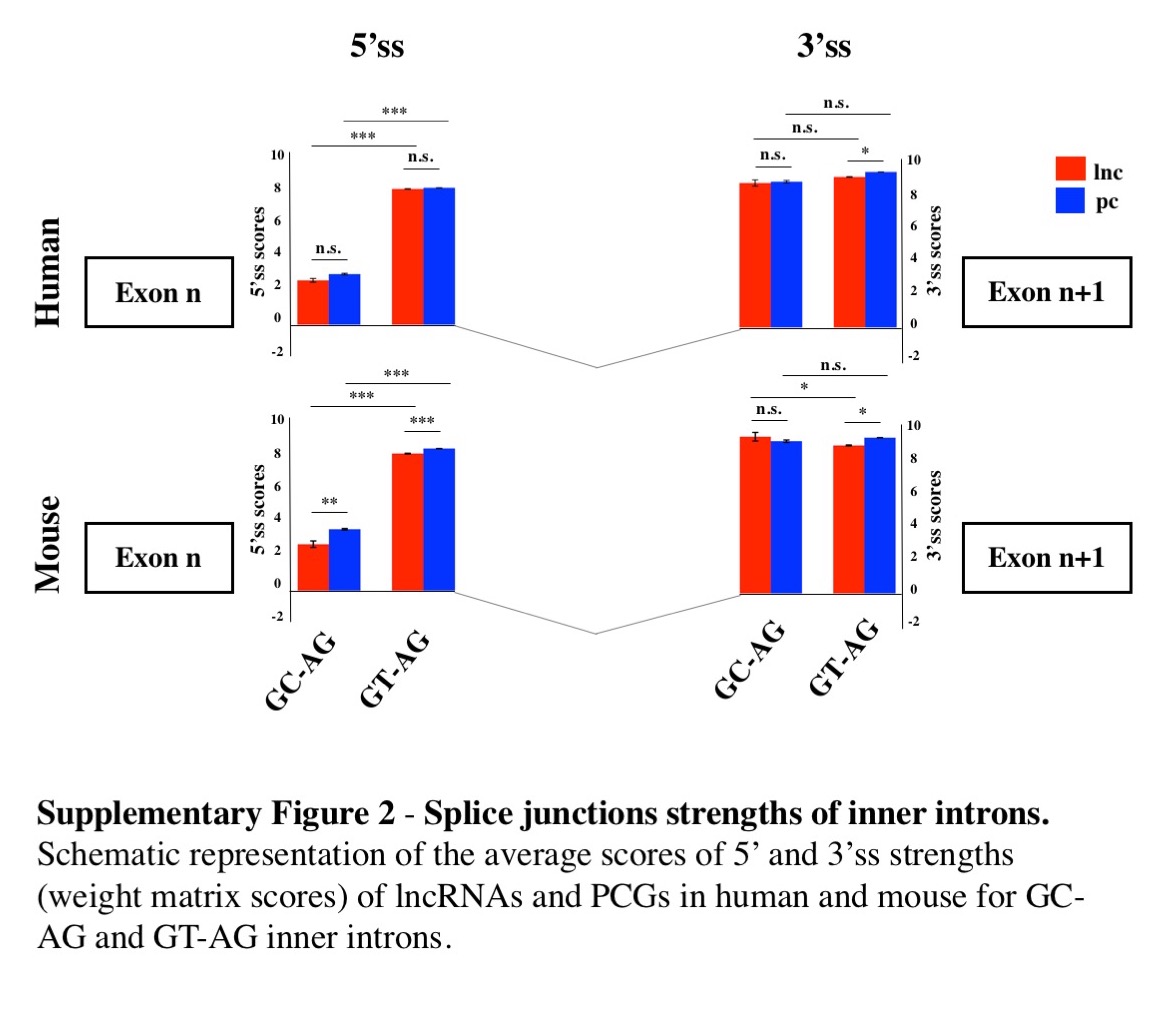

Supplement: Supplementary file 3 [file Image_2.JPEG]

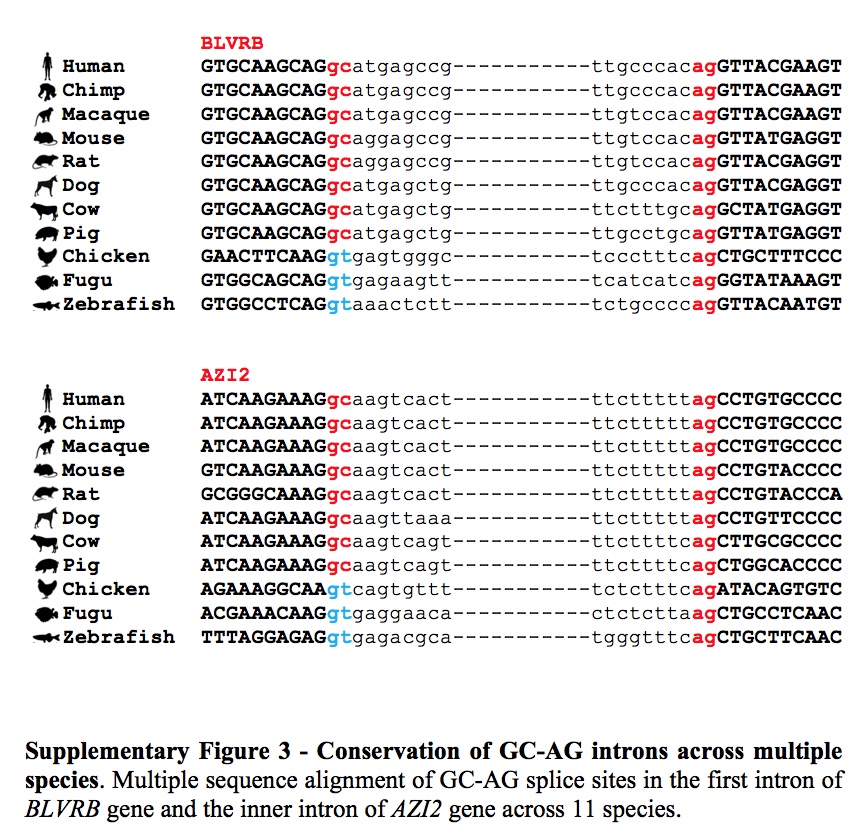

Supplement: Supplementary file 4 [file Image_3.JPEG]
