# Supplementary material for: GC-AG Introns Features in Long Non-coding and Protein-Coding Genes Suggest Their Role in Gene Expression Regulation
Source: Front Genet. 2020 May 15;11:488. doi: 10.3389/fgene.2020.00488 (PMC7242645; doi:10.3389/fgene.2020.00488)
Supplement: Supplementary file 5 [file Image_4.pdf]

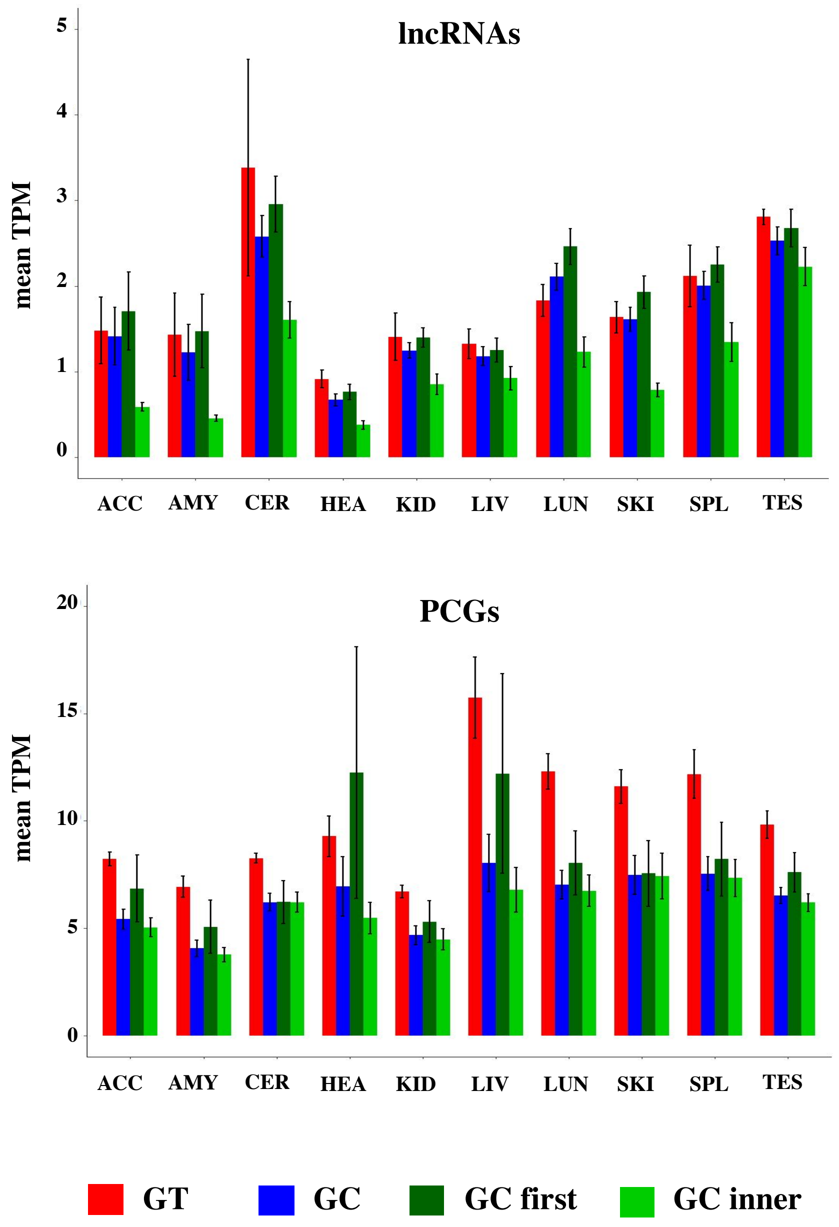


**Figure 4 – Expression of GC-AG- and GT-AG-containing transcripts.** Bar graph representing the expression of lncRNAs and PCGs transcripts in different human tissues (acc: anterior cingulate cortex; amy: amygdala; cer: cerebellum; hea: heart; kid: kidney; liv: liver; lun: lung; ski: skin; spl: spleen; tes: testis)Transcripts were divided as containing GC-AG- or GT-AG-introns and between transcripts containing a GC-AG intron in the first or inner position. The expression of transcripts was calculated as mean TPM combining expression data from 10 different tissues together.
